# Oral Preparation of Hyaluronic Acid, Chondroitin Sulfate, Curcumin, and Quercetin (Ialuril^®^ Soft Gels) for the Prevention of LUTS after Intravesical Chemotherapy

**DOI:** 10.3390/pathophysiology29030028

**Published:** 2022-07-13

**Authors:** Celeste Manfredi, Lorenzo Spirito, Francesco Paolo Calace, Raffaele Balsamo, Marco Terribile, Marco Stizzo, Lorenzo Romano, Luigi Napolitano, Gianluigi Califano, Luigi Cirillo, Giovanni Maria Fusco, Claudia Rosati, Carmelo Quattrone, Carmine Sciorio, Massimiliano Creta, Nicola Longo, Marco De Sio, Davide Arcaniolo

**Affiliations:** 1 Urology Unit, Department of Woman, Child and General and Specialized Surgery, University of Campania “Luigi Vanvitelli”, 80131 Naples, Italy; manfredi.celeste@gmail.com (C.M.); lorenzospirito@msn.com (L.S.); marcostizzo@hotmail.com (M.S.); carmeloquattrone@hotmail.it (C.Q.); marco.desio@unicampania.it (M.D.S.); davide.arcaniolo@gmail.com (D.A.); 2 Urology Unit, AORN Ospedali dei Colli, Monaldi Hospital, 80131 Naples, Italy; raffaelebalsamo5@gmail.com; 3 Urology Unit, Clinica Trusso, 80044 Naples, Italy; mrcterribile@gmail.com; 4 Urology Unit, Department of Neurosciences, Reproductive Sciences, and Odontostomatology, University of Naples “Federico II”, 80131 Naples, Italy; loryromano@hotmail.it (L.R.); luiginap89@gmail.com (L.N.); gianl.califano2@gmail.com (G.C.); cirilloluigi22@gmail.com (L.C.); giom.fusco@gmail.com (G.M.F.); max.creta@gmail.com (M.C.); nicola.longo@unina.it (N.L.); 5 Department of Clinical Medicine and Surgery, University of Naples “Federico II”, 80131 Naples, Italy; clarosat@unina.it; 6 Department of Urology, ASST Lecco, Ospedale Alessandro Manzoni, 23900 Lecco, Italy; carmine.sciorio@gmail.com

**Keywords:** hyaluronic acid, oral formulation, intravesical chemotherapy, LUTS

## Abstract

Intravesical chemotherapy may cause chemical cystitis and related lower urinary tract symptoms (LUTS). The aims of this study were to evaluate the efficacy and safety of an oral preparation of hyaluronic acid (HA), chondroitin sulfate (CS), curcumin, and quercetin (Ialuril^®^ Soft Gels) to reduce the severity of LUTS in patients with a history of bladder cancer (BCa) undergoing intravesical chemotherapy. We designed a monocentric, randomized, double-blind, placebo-controlled pilot trial. Patients referred to our institute between November 2016 and March 2018 were enrolled. All subjects had non-muscle-invasive BCa and received intravesical chemotherapy with mitomycin C (MMC). Patients were randomized 1:1 in two groups (intervention vs. control). All subjects underwent oral administration (Ialuril^®^ Soft Gels or placebo) starting one week before the first weekly instillation and ending 30 days after the last one, subsequently starting one week before each monthly instillation and ending 14 days after it. International prostate symptom score (IPSS) and 0-100 visual analogue scale (VAS) were used to assess the efficacy of the treatment. Adverse events were also described. Patients were evaluated at baseline and after 1, 4, 7, and 13 months of intravesical chemotherapy. A total of 34 patients were enrolled. The median IPSS score was significantly lower in the intervention group compared to the control group at 4 (13 vs. 17 points; *p* = 0.038), 7 (10 vs. 18 points; *p* < 0.001), and 13 (10 vs. 17 points; *p* = 0.002) months. The median VAS score was significantly lower in the intervention group compared to the control group at 7 (22 vs. 37 points; *p* = 0.021) and 13 (20 vs. 35 points; *p* = 0.024) months. No AE specifically related to supplement or placebo was recorded. Oral formulation of HA, CS, quercetin, and curcumin could be an effective and safe supportive therapy against chemical cystitis in patients receiving intravesical chemotherapy for BCa.

## 1. Introduction

Bladder cancer (BCa) is the 7th most commonly diagnosed tumor in the male population worldwide, being 10th when both genders are taken into account. The worldwide age-standardized incidence rate (per 100,000 person/years) is 9.5 and 2.4 in men and women, respectively [1]. Smoking is the most important risk factor for BCa, accounting for approximately 50% of cases [2,3]. Urothelial carcinoma corresponds to approximately 90% of all bladder tumors [4]. The diagnosis of BCa is based on cystoscopy and histological evaluation of sampled tissue by either cold-cup biopsy or, most commonly, resection with transurethral resection of bladder (TURB). The objective of TURB is to make the correct histological diagnosis, perform local staging, and completely remove all visible lesions [5]. Transurethral resection of bladder (TURB) can eradicate a Ta/T1 tumor completely, although these lesions commonly recur and progress to muscle-invasive cancers, which makes intravesical adjuvant therapy a key intervention in this setting [6,7].

The type of adjuvant treatment after TURB should be based on the risk of recurrence. In patients at low recurrence risk and in selected patients at intermediate risk, one immediate instillation of chemotherapy is recommended. In subjects with intermediate-risk tumors, one-year full-dose intravesical Bacillus Calmette-Guérin (BCG) or instillations of chemotherapy for a maximum of one year is recommended. In patients at high risk, full-dose intravesical BCG for one to three years is indicated [5].

Intravesical chemotherapy may cause systemic side effects due to absorption through the bladder epithelium; however, local toxicity is more common [8]. Administration of cytotoxic chemotherapy into the bladder can induce dysuria, frequency, urgency, suprapubic discomfort, gross hematuria, and pelvic pain [9]. These symptoms are collectively referred to a chemical cystitis, which has an incidence of about 10% [10,11]. Some patients may not complete the scheduled treatment due to severe lower urinary tract symptoms (LUTS), with a possible worsening in their oncological outcomes [10]. Moreover, LUTS seem to be an important factor associated with the impairment in the quality of life (QoL) of these patients [12].

The bladder urothelium provides a protective barrier against the penetration of toxic agents, urine, and bacteria. The glycosaminoglycan (GAG) layer consists of a thick mucus layer of glycoproteins and proteoglycans on the surface of the urothelial cells [13]. The main components of this superficial layer are chondroitin sulfate (CS) and hyaluronic acid (HA). Damage to the GAG layer, consequent to several pathological processes, alters its protective barrier function, leading to increased permeability into the deep layers of the urothelium, and, ultimately, it may cause urgency, frequency, and pain [14].

The intravesical and oral administration of HA and CS were proposed to restore the GAG layer and consequently to improve the symptoms in patients with bladder pain syndrome, recurrent bacterial cystitis, radiation, and chemical cystitis [15,16]. Although several supplements have been positively evaluated in the context of inflammatory diseases of the genitourinary tract [17], currently, there are no studies investigating the impact of the oral administration of these molecules in patients undergoing intravesical chemotherapy.

The primary aim of the study was to evaluate the efficacy of an oral preparation of HA, CS, quercetin, and curcumin after intravesical chemotherapy for non-muscle-invasive BCa to reduce the severity of associated LUTS. The secondary objective was to assess adverse events related to the oral preparation in the same setting.

## 2. Materials and Methods

### 2.1. Study Design

We designed a monocentric, randomized, double-blind, placebo-controlled pilot trial. It was approved by the Ethical Committee of University of Campania “Luigi Vanvitelli” (n.519–03/05/16) and conducted according to the Declaration of Helsinki on ethical principles for medical research involving human subjects.

Patients referred to our center between November 2016 and March 2018 were enrolled after providing written informed consent, including acceptance of data publication. At the time of enrollment, patients were randomized with a 1:1 allocation ratio by means of a computer-generated random list in two groups (intervention and control).

### 2.2. Inclusion and Exclusion Criteria

All patients were at least 18 years old, had a history of previous TURB with diagnosis of non-muscle-invasive BCa, and had been scheduled for intravesical chemotherapy according to current guidelines [5].

Patients with the following characteristics were excluded: muscle-invasive BCa; carcinoma in situ (CIS); intravesical immunotherapy with Bacillus Calmette-Guérin (BCG); concomitant urinary tract infections; urinary tract abnormalities or dysfunctions; use of other supplements that contain similar active principles or that may interfere with study evaluations; and any absolute contraindication to the drugs or supplements used in the study.

### 2.3. Patient Assessment and Data Collection

Patients were evaluated at baseline and after 1, 4, 7, and 12 months of intravesical chemotherapy.

At baseline, the following data were collected: demographic information, medical history, physical examination, urinalysis, urine culture, and ultrasonography of the urinary tract. Type (primary or recurrent), focality, histology, grading, and pathological stage of BCa were recorded. Post-void residual urine volume (PVR), peak flow rate (Qmax), international prostate symptom score (IPSS), and 0–100 visual analogue scale (VAS) for bother from LUTS were self-administered to the patients during the baseline visit. Urinalysis, urine culture, IPSS, and VAS were repeated at the time of follow-up visits. Standard IPSS was used for both the men and women enrolled. VAS ranged from 0 mm (no bother) to 100 mm (worst bother imaginable). All adverse events (AEs) during the study period were recorded.

### 2.4. Treatment Protocol

An oral preparation (capsule) of HA 20 mg, CS 200 mg, quercetin 200 mg, curcumin 200 mg (Ialuril^®^ Soft Gels, IBSA Farmaceutici, Lodi, Italy), and an intravesical preparation of mitomycin C (MMC) 40 mg were used for the study.

Intravesical chemotherapy was administered following the protocol of 1 instillation per week for the first month and 1 instillation per month for the subsequent 12 months. A single-shot instillation immediately after surgery was also always performed [5].

The oral preparation (supplement or placebo) was administered according to the following scheme: 2 capsules taken at the same time in the morning, starting one week before the first weekly instillation and ending 30 days after the last one, and subsequently, 2 capsules at the same time in the morning, starting one week before each monthly instillation and ending 14 days after it.

### 2.5. Statistics

The median was used as measure of central tendency and interquartile range (IQR) as measure of statistical dispersion. The Shapiro–Wilk test was performed as the normality test. The Wilcoxon (paired or unpaired) and chi-squared tests were used to assess differences between the two groups and in the same group at different follow-up visits. Statistical significance was arbitrarily set for a *p*-value < 0.05. The IBM Statistical Package for the Social Sciences (IBM Corp. Released 2015. IBM SPSS Statistics for Windows, Version 23.0. Armonk, NY, USA, IBM Corp.) was used for the statistical analyses.

## 3. Results

**Figure 1:**
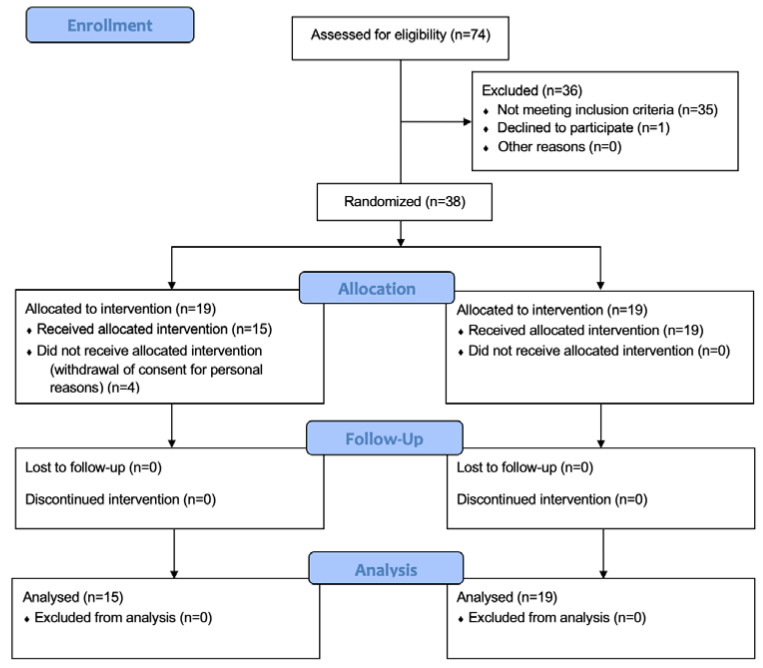
Consort 2010 Flow Diagram.

A total of 34 patients were available for the analysis: 19 in the placebo group and 15 in the supplement group (Figure 1). The baseline characteristics were similar between the two groups (Table 1). All bladder tumors were non-muscle-invasive urothelial carcinomas. No significant difference (*p* > 0.05) was found in type, focality, grading, and pathological stage of BCa between the two groups (Table 2). All patients in both groups completed the scheduled visits.

**Figure 2:**
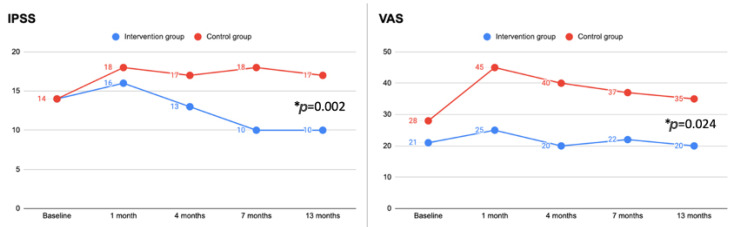
IPSS and VAS during the study period. All patients in both groups completed the scheduled visits (intervention group *n* = 15; control group *n* = 19). Values are reported as medians. IPSS: international prostate symptom score. VAS: visual analogue scale (0–100). * Referred to the last follow-up visit (13 months).

The median IPSS score was significantly lower in the intervention group compared to the control group at 4 (13 vs. 17 points; *p* = 0.038), 7 (10 vs. 18 points; *p* < 0.001), and 13 (10 vs. 17 points; *p* = 0.002) months. Compared to baseline, a significant improvement (*p* < 0.05) in the IPSS score was reported for the intervention group at 7 and 13 months, while a significant worsening (*p* < 0.05) in the IPSS score was found for the control group at each follow-up visit (Table 3). The median VAS score was significantly lower in the intervention group compared to the control group at 7 (22 vs. 37 points; *p* = 0.021) and 13 (20 vs. 35 points; *p* = 0.024) months. Compared to baseline, no significant difference (*p* > 0.05) in the VAS score was reported for the intervention group at each follow-up control, while a significant worsening (*p* < 0.05) in the VAS score was found for the control group at each follow-up visit (Table 4). The trend in IPSS and VAS during the follow-up in both groups is shown in Figure 2. IPSS and VAS scores at the last follow-up were confirmed to be significantly improved (*p* < 0.05) in the intervention group compared to the control group when analyzing men and women separately. No significant differences (*p* > 0.05) were found in the IPSS and VAS scores between groups on the basis of age. Median IPSS-quality of life (IPSS-QoL) score at baseline was not significantly different between the two groups (*p* = 0.088). During the last follow-up, IPSS-QoL was significantly better in the intervention group (5.0 vs. 3.0; *p* = 0.021). No AE specifically related to supplement or placebo was recorded. Dysuria was significantly less frequent (*p* = 0.029) in the intervention group. Local side effects of intravesical chemotherapy were reported in Table 5. Systemic AEs related to intravesical chemotherapy were not described.

## 4. Discussion

Chemical cystitis can be caused by intravesical chemotherapy. Patients receiving intravesical MMC can suffer from local side effects (24%), with a cystitis rate of approximately 10% [18]. Although chemical cystitis and the associated LUTS can have a substantial impact on QoL and treatment compliance, there is no established supportive therapy to prevent it [11]. Some evidence suggests that the intravesical administration of HA may be effective in preventing chemical cystitis. In a preclinical mouse model of chemical cystitis induced by administering hydrogen chloride (400 microL, 10 mM), a single dose of intravesical HA (0.5 mL, 0.8 mg/mL) was associated with a drop in local levels of inflammation [19]. Additionally, the combined intravesical administration of HA and CS appears to be beneficial in patients with BCG-associated chemical cystitis, as shown in a retrospective study by Imperatore et al. [15]. In this article, including 20 patients with BCG-induced chemical cystitis and treated with eight weekly instillations of HA/CS, the mean (SD) VAS scores for urinary urgency and bladder pain decreased significantly from 7.8 ± 0.5 and 7.2 ± 1.0 at baseline to 4.7 ± 1.1 and 4.2 ± 0.9 at the end of treatment (*p* < 0.05 in both cases). Considering these encouraging findings obtained with intravesical instillations, we hypothesized that an oral formulation containing HA, CS, and other carefully selected natural ingredients could be beneficial for the treatment and prevention of chemical cystitis and associated LUTS. The formulation tested in our trial also included curcumin—which represents the most abundant polyphenol found in the rhizome of *Curcuma longa* [20] and expresses strong anti-inflammatory effects due to its antioxidant capacity—as well as quercetin—one of the most studied flavonols, which is abundant in fruits and vegetables and acts as an enhancer of curcumin intestinal absorption and bioavailability, also exerting an intrinsic inflammatory activity [21,22]. Even in the small patient population considered, we found that oral preparation of HA, CS, curcumin, and quercetin could promote a significant improvement in LUTS in patients undergoing intravesical chemotherapy, in terms of both IPSS and VAS. Moreover, we reported no AE specifically related to the supplement. Our findings are consistent with those reported in the small randomized controlled trial (RCT) without placebo by Redorta et al. The authors showed that intravesical HA/CS plus the oral administration of HA, CS, curcumin, and quercetin was associated with reduced bladder toxicity in patients undergoing radiotherapy for prostate cancer [23]. The composition, dosage, and hypothesized mechanism of action of Ialuril^®^ Soft Gels components are summarized in Table 6.

To the best of our knowledge, this is the first RCT on the role of oral preparation of HA, CS, curcumin, and quercetin as supportive therapy against chemical cystitis and the accompanying LUTS in patients with BCa receiving intravesical chemotherapy. The main strength of our study is the rigorous methodology with a randomized, double-blind, placebo-controlled design. However, our trial presents several limitations. First of all is the small sample size; however, it should be considered as a pilot trial in order to evaluate the feasibility of the study in a larger cohort of patients. Neither the pharmacokinetic profile of the administered compounds nor the oncological outcomes were evaluated; however, these were not among the aims of our study. The efficacy and safety of the individual components of the oral formulation were not evaluated separately; therefore, it was not possible to determine which compounds were most responsible for the results described. Similarly, the possible drug interaction of the single components between them and with other drugs was not studied. The small increase in the IPSS score of the control group during follow-up was unexpected, limiting the interpretability of the data. Finally, the administration protocol was not standardized, but as there were no previous studies in the literature, this was inevitable.

## 5. Conclusions

In conclusion, based on our preliminary data, oral formulation of HA, CS, quercetin and curcumin could be an effective and safe supportive therapy against chemical cystitis and the concomitant LUTS in patients with non-muscle-invasive BCa receiving intravesical MMC. Further well-designed RCTs with a larger sample size and longer follow-up are needed to confirm our encouraging results.

## Figures and Tables

**Table 1:** Baseline characteristics of the patients.

	Intervention Group (*n* = 15)	Control Group (*n* = 19)	*p*-Value
***Age***			0.082
*Median* *(IQR)*	73 (65–78.5)	69 (61–73.5)	
***Gender*** *N (%)*			0.118
*Males* *Females*	13 (86.7) 2 (13.3)	15 (79.0) 4 (21.0)	
***VAS***			0.103
*Median* *(IQR)*	21.0 (6.7–41.0)	28.0 (7.5–59.0)	
***IPSS***			0.061
*Median* *(IQR)*	14.0 (8.0–16.7)	14.0 (8.5–18.0)	
***IPSS-QoL***			0.088
*Median* *(IQR)*	3 (1.0–4.0)	3 (2.0–4.0)	
***Qmax***			0.213
*Median* *(IQR)*	13.5 (7.2–19.4)	14.1 (8.1–18.9)	
***PVR***			0.191
*Median* *(IQR)*	10.0 (0–15.0)	7.0 (3.7–12.5)	

VAS: visual analogue scale. IPSS: international prostate symptom score. Qmax: peak flow rate. PVR: post-void residual urine volume. IQR: interquartile range. QoL: quality of life.

**Table 2:** Bladder tumors’ characteristics.

	Intervention Group (*n* = 15)	Control Group (*n* = 19)	*p*-Value
***Type*** *N (%)*			0.012
*Primary* *Recurrent*	13 (86.7) 2 (13.3)	16 (84.2) 3 (15.8)	
***Focality*** *N (%)*			0.233
*Monofocal* *Multifocal*	8 (53.3) 7 (46.7)	10 (53.6) 9 (47.4)	
***Histotype*** *N (%)*			0.087
*Urothelial* *Non-urothelial*	15 (100) 0 (0)	19 (100) 0 (0)	
***Grading*** *N (%)*			0.122
*Low-grade* *High-grade*	11 (73.3) 4 (26.7)	15 (79.0) 4 (21.0)	
***Pathologic Stage*** *N (%)*			0.093
*Ta* *T1*	9 (60.0) 6 (40.0)	12 (63.2) 7 (36.8)	
***EAU Risk group*** *N (%)*			0.321
*Low risk* *Intermediate risk*	12 (80) 3 (20)	14 (73.7) 5 (26.3)	

EAU: European Association of Urology.

**Table 3:** IPSS in the two groups at different time points (median, IQR).

	Baseline	1 Month	4 Months	7 Months	13 Months	*p*-Value *
*Intervention group (n = 15)*	14.0 (8.0–16.7)	16 (9–18.5)	13 (9.2–17.5)	10 (9–15)	10 (8–16)	0.067 ^a^ 0.322 ^b^ **0.001 ^c (I)^** **0.001 ^d (I)^**
*Control group (n = 19)*	14.0 (8.5–18.0)	18 (13–23.7)	17 (14.2–22.7)	18 (14–22.7)	17 (14–21.5)	**<0.001 ^a (W)^** **0.026 ^b (W)^** **<0.001 ^c (W)^** **0.003 ^d (W)^**
p-value **	0.061	0.139	**0.038**	**<0.001**	**0.002**	

All patients in both groups completed the scheduled visits. * Intragroup analysis (Baseline vs. 1 month ^a^, Baseline vs. 4 months ^b^, Baseline vs. 7 months ^c^, Baseline vs. 13 months ^d^). ** Intergroup analysis (Intervention group vs. Control group) (I): Improvement; (W): Worsening. IPSS: international prostate symptom score. IQR: interquartile range.

**Table 4:** 0–100 VAS in the two groups at different time points (Median, IQR).

	Baseline	1 Month	4 Months	7 Months	13 Months	*p*-Value *
*Intervention group (n = 15)*	21.0 (6.7–41.0)	25 (16.2–45.7)	20 (11.2–38.75)	22 (8.5–37.25)	20 (10–29.2)	0.342 ^a^ 0.054 ^b^ 0.077 ^c^ 0.328^d^
*Control group (n = 19)*	28.0 (7.5–59.0)	45 (20.5–67.2)	40 (17–75.7)	37 (17.7–67.2)	35 (15.7–60)	**<0.001 ^a (W)^** **<0.001 ^b (W)^** **0.001 ^c (W)^** **0.001 ^d (W)^**
p-value **	0.103	0.145	0.092	**0.021**	**0.024**	

All patients in both groups completed the scheduled visits. * Intragroup analysis (Baseline vs. 1 month ^a^, Baseline vs. 4 months ^b^, Baseline vs. 7 months ^c^, Baseline vs. 13 months ^d^). ** Intergroup analysis (Intervention group vs. Control group) (I): Improvement; (W): Worsening. VAS: visual analogue scale. IQR: interquartile range.

**Table 5:** Local side effects of intravesical chemotherapy.

Local Adverse Event	Intervention Group (*n* = 15)	Control Group (*n* = 19)	*p*-Value
***Hematuria*** *N (%)*	3 (20.0)	5 (26.3)	0.078
***Dysuria*** *N (%)*	4 (26.7)	7 (36.8)	**0.029**
***Urinary tract infection*** *N (%)*	0 (0)	1 (5.3)	0.433

**Table 6:** Ialuril^®^ Soft Gels: components, dosages, and mechanisms of action.

Component	Dosage	Mechanism of Action
*HA*	20 mg	To restore the GAG layer on the urothelial surface of the bladder
*CS*	200 mg	To restore the GAG layer on the urothelial surface of the bladder
*Quercetin*	200 mg	Anti-inflammatory effects due to antioxidant capacity
*Curcumin*	200 mg	Anti-inflammatory effects due to antioxidant capacity

HA: hyaluronic acid; CS: chondroitin sulfate; GAG: glycosaminoglycan. All components are present in a single capsule.

## Data Availability

The authors declare that data are available on request.
